# Enhancing Emotion Recognition Using Region-Specific Electroencephalogram Data and Dynamic Functional Connectivity

**DOI:** 10.3389/fnins.2022.884475

**Published:** 2022-05-02

**Authors:** Jun Liu, Lechan Sun, Jun Liu, Min Huang, Yichen Xu, Rihui Li

**Affiliations:** ^1^College of Information Engineering, Nanchang Hangkong University, Nanchang, China; ^2^College of Aviation Service and Music, Nanchang Hangkong University, Nanchang, China; ^3^Department of Psychiatry and Behavioral Sciences, Center for Interdisciplinary Brain Sciences Research, Stanford University, Stanford, CA, United States

**Keywords:** sequential backward feature selection, Xception architecture, emotion recognition, EEG channel selection, dynamic functional connectivity

## Abstract

Recognizing the emotional states of humans through EEG signals are of great significance to the progress of human-computer interaction. The present study aimed to perform automatic recognition of music-evoked emotions through region-specific information and dynamic functional connectivity of EEG signals and a deep learning neural network. EEG signals of 15 healthy volunteers were collected when different emotions (high-valence-arousal vs. low-valence-arousal) were induced by a musical experimental paradigm. Then a sequential backward selection algorithm combining with deep neural network called Xception was proposed to evaluate the effect of different channel combinations on emotion recognition. In addition, we also assessed whether dynamic functional network of frontal cortex, constructed through different trial number, may affect the performance of emotion cognition. Results showed that the binary classification accuracy based on all 30 channels was 70.19%, the accuracy based on all channels located in the frontal region was 71.05%, and the accuracy based on the best channel combination in the frontal region was 76.84%. In addition, we found that the classification performance increased as longer temporal functional network of frontal cortex was constructed as input features. In sum, emotions induced by different musical stimuli can be recognized by our proposed approach though region-specific EEG signals and time-varying functional network of frontal cortex. Our findings could provide a new perspective for the development of EEG-based emotional recognition systems and advance our understanding of the neural mechanism underlying emotion processing.

## Introduction

Emotion is present in all aspects of human life and an important support for human communication and exchange. Emotion can have a significant impact on decision making and judgment (Gupta et al., 2019), and are very closely related to consciousness (Hasanzadeh et al., 2021). Accurate perception of emotions is of great importance for social communication, while incorrect recognition of emotion states may lead to interpersonal communication difficulty (Zinchenko et al., 2017). Besides, emotion recognition drives the development of human-computer interaction (HCI) systems and occupies an important position in the field of human interaction (Li P. et al., 2019). Therefore, increasing attention has been paid to automatic emotion recognition systems, which cannot only improve the performance of HCI system, but also provides a basis for further exploration of neural mechanism underlying emotion processing and regulation.

Given the importance of emotion recognition, a growing number of researchers are conducting in-depth exploration on this field. Recognition of emotion are usually divided into two categories: physiological signals and non-physiological signals, depending on how signal is collected. Non-physiological signals include expressions (Liu et al., 2017; Liliana, 2019), body postures (Noroozi et al., 2021), voice signals (Wang et al., 2020), etc., while physiological signals include electroencephalogram (EEG) (Namazi et al., 2020; Song et al., 2020), electrocardiogram (ECG) (Al-Sheikh et al., 2019; Nguyen et al., 2019; Fang et al., 2020; Sarkar and Etemad, 2021) and electromyography (EMG) (Hossen et al., 2020; Kulke et al., 2020), etc. Among the various physiological signals, EEG signals have attracted widespread attention due to its high mobility and close relationship with neural response (Li et al., 2017, 2020b). With the rapid development of dry electrode technology, portable and low-cost EEG devices are gradually gaining popularity among researchers, and EEG-based emotion recognition has also been increasingly explored in more and more studies (Gupta et al., 2019; Taran and Bajaj, 2019).

How to induce emotions and classify different emotional states have been challenges for many EEG-based emotion recognition studies. The most common method of emotion induction is the presentation of emotional materials. Ahirwal and Kose (2019) conducted a study on emotion recognition and physiological arousal by presenting video stimuli to the subjects. The effect of different picture interference on the emotion recognition was investigated by presenting picture stimuli in a previous study (Wang et al., 2018). In addition, as the art of directly expressing human emotions, music can also be used as stimulus presentations. According to a previous study, music stimuli could cause changes in the main counter-components of emotions such as autonomic and endocrine responses, thus evoking real emotions (Koelsch, 2014). In this context, music and music video have been thought to be more profound compared with other materials (Suhaimi et al., 2020).

In terms of emotion recognition, numerous EEG-based studies have been performed in the past few decades to achieve this goal. Among them, a variety of EEG features and machine learning-based classification techniques have been extensively investigated to illustrate the specificity of brain activity associated with different emotional states and to enhance the performance of emotion recognition (Mauss and Robinson, 2009; Lin et al., 2010; Jenke et al., 2014; Bo et al., 2018). However, a major limitation of these approaches is that most existing studies have simply focused on EEG characteristics extracted from single or whole-brain electrode channels separately. This type of analyses failed to take advantage of the region-specific neuronal information, or the spatiotemporal-varying interactions at the network level (e.g., cluster, time-varying pattern) to allow a deeper understanding of the brain-emotion relationship. As indicated in a previous study, emotion processing and regulation is likely to involve complex neural circuits in a time-varying manner rather than any independent brain region (Mauss and Robinson, 2009; Fang et al., 2020). Besides, various studies have reported that the frontal cortex seems to play a more essential role in emotion-related activity compared to other brain regions such as temporal, parietal, and occipital (Sarno et al., 2016). Taken these together, analysis approaches that examine time-varying regional interaction of frontal cortex, as well as network-level coupling among multiple brain sites may hold great promise for understanding neural signatures associated with different emotion states and improving the accuracy of automatic emotion recognition.

In this study, we proposed a deep learning-based approach to enhance the performance of an EEG-based emotion recognition system. Specifically, we first evaluated whether using a subset of EEG channels in frontal cortex could help improve the classification performance, from which an optimal EEG channel combination for emotion recognition was obtained. Furthermore, we assessed how the dynamic functional network of frontal cortex may affect the classification performance through a trial-by-trial, time-varying manner. The contributions of the present study include: (1) we proposed a method to convert spatial information of multi-channel EEG signals to 2D image matrix as inputs of deep learning neural network; (2) we provided evidence supporting that frontal cortex, particularly part of the frontal cortex, plays an essential role in emotion recognition, through the combination of a deep learning-based technique and a sequential feature selection algorithm, and (3) we explored how time-varying functional connectivity within the frontal cortex may affect the performance of emotion recognition.

## Materials and Methods

### Subjects

A total of 15 volunteers (14 males and 1 female, age: 20.4 ± 2.16 years) were recruited for the EEG-based emotion recognition under high valence-arousal (HVA) and low valence-arousal (LVA) musical stimuli. None were musicians or have working experience in music-related industries. All subjects were right-handed, had no hearing impairment, no neurological history. Within 48 h before the experiment, subjects were informed to keep adequate sleep, and not to smoke, drink alcohol or functional beverages. This study is approved by the Institutional Review Board of Nanchang Hangkong University. Informed consent was obtained from all participants included in the study.

### Data Acquisition

**FIGURE 1:**
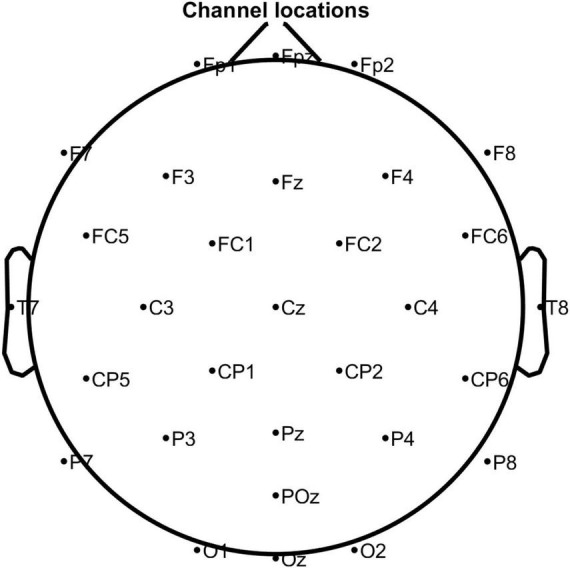
The position of the electrodes according to the International 10–20 system. Note that M1 and M2 were used as reference electrodes.

In this study, the EEG system (eego mylab, Ant Neuro, Netherlands) was used to collect 32-channel EEG signals with a sampling frequency of 500 Hz. The electrode positions were placed according to the international 10–20 system (Figure 1). A presentation software (E-Prime 3.0, Psychology Software Tools, American) was used to design and present the experimental stimuli.

### Experimental Protocol

**FIGURE 2:**
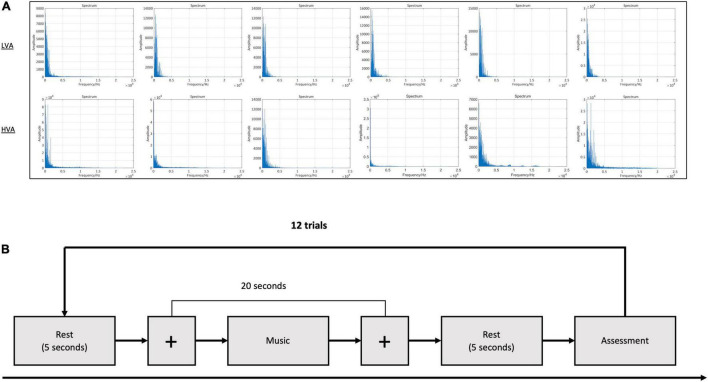
The experimental protocol. **(A)** Power spectrum of the music clips. **(B)** The experimental paradigm of music-evoked emotions.

Twelve music clips were selected by a music expert as stimuli, of which six each were used to stimulate the production of HVA and LVA emotions, respectively. Table 1 shows the detailed information of each music clip, including performer, name, and time duration of the clip. We also calculated the averaged power spectrum of each type of music stimuli. As shown in Figure 2A, the power distribution of all selected music clips was mainly located at very low frequency. During the experiment, each subject was presented with 12 trials, wherein each trial contained three parts: a 5-s resting period, a 20-s stimulation period, and a 5-s resting state. Participants were requested to focus on a visual fixation to reduce eyes movement during the stimulation period (Figure 2B; Bo et al., 2018). At the end of each trial, the subjects were asked to perform a self-assessment of emotional valence and arousal. Non-invasive EEG signals are highly susceptible to external signals that produce noise and artifacts. Common sources of interference include 50 Hz AC, ocular and myoelectricity, and other high-power devices. To reduce the influence of interference sources, we adopted the following experimental setup. First, the experimental environment was optimized to ensure the reliability of the data. Specifically, the experiment was conducted in a recording booth with suitable temperature, humidity, and light, and a comfortable seat was provided for the subjects. Second, cell phones and other wireless devices were required to be off throughout the experiment, and verbal communication between the subjects and the researcher was prohibited during the experiment.

**TABLE 1:** Detailed information of all music clips.

Classification	Performer	Name of music clip	Duration (mm:ss)
LVA	Richard Clayderman	A comme amour (L for love)	0:00–0:20
LVA	Yiruma	Kiss the rain	0:50–1:10
LVA	Kevin Kern	In the enchanted garden (Sundial dreams)	0:10–0:30
LVA	The Daydream	Dreaming (Tears)	0:00–0:20
LVA	Kevin Kern	In the enchanted garden (Through the arbor)	0:05–0:25
LVA	Jin Shi	Melody of the night (Five)	0:13–0:33
HVA	Hiphop	My view (A little story)	0:18–0:38
HVA	Fryderyk Franciszek Chopin	Chopin’s revolutionary etude in c minor	0:22–0:42
HVA	Richard Clayderman	Ballade pour Adeline (Mariage d’amour)	1:42–2:02
HVA	July	My soul	0:25–0:45
HVA	Wolfgang Amadeus Mozart	Alla turca	1:33–1:53
HVA	Richard Clayderman	Lyphard melodie	1:07–1:27

### Data Analysis

Majority of previous studies have focused purely on the effect of independent channel EEG power on classification results, which neglected the spatial relationships between channels (Tang et al., 2019; Raghu et al., 2020). To address this problem, in this study, we first proposed a sequential backward selection algorithm, combined with Xception neural network, to perform enhanced classification of binary emotion states as well as identify the spatially optimal EEG channel combinations. In addition, we analyzed the detailed difference of regional network between two emotional states using coherence-based functional network analysis. In particular, we explored how the dynamic alteration of functional network may affect the classification performance of two emotions.

#### Preprocessing of Electroencephalogram Data

The preprocessing of EEG data was performed using EEGLAB (Delorme and Makeig, 2004) and customized MATLAB script. First, the EEG signals were filtered with a notch filter (49–51 Hz) and a bandpass filter (1–40 Hz) to remove powerline interference and other noises. The common average reference (CAR) method was used to re-reference the multi-channel EEG data (Yao et al., 2019). We then used independent component analysis (ICA) to remove the interference of electrooculography (EOG). To ensure the completeness of data, no data exclusion was done on segments with artifact (e.g., large spike). In fact, no obvious large artifacts were identified through visual inspection of the preprocessed EEG signals. Single trial EEG data were finally corrected for baseline and segmented for further analysis.

#### Training of Neural Network Model

**FIGURE 3:**
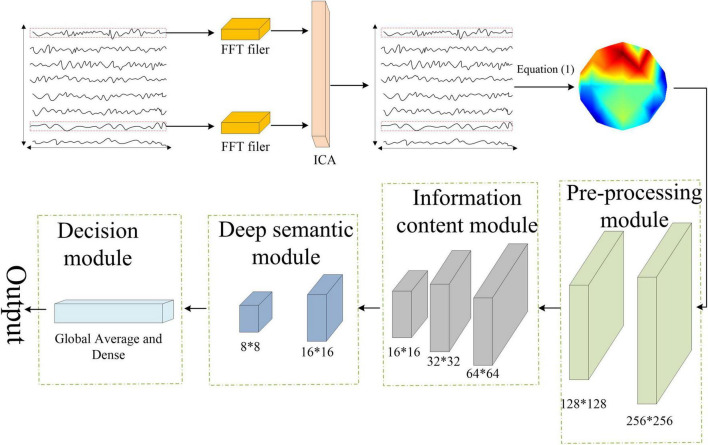
The analysis pipeline of the proposed Xception network-based emotion recognition.

In recent years, various neural networks developed based on deep learning have shown superior performance in neuroimaging studies compared to traditional machine learning techniques. In this study, we used Xception network to extract image-based features (Xu et al., 2019; Liu et al., 2021; Peng et al., 2021). The Xception model is based on depth-separable convolution, and its structure consists of 14 residual modules composed of 3 common convolutional layers and 33 depth-separable convolutional ones. The common convolution is contained in the preprocessing module, as shown in Figure 3.

The classification process can be roughly divided into four steps. First, the preprocessed EEG signal was sampled per second, and the spatial coordinate information of each sampling point was recorded. Next, the spatial information of the sampled EEG data was converted into an RGB image using Equation (1).


(1) $${\text{Y}}_{i\,j}=\sum \sum \frac{\left(X_{i\,j}-\min\left(X\right)\right)*255}{\max\left(X\right)-\min\left(X\right)}$$

where X is the matrix containing the EEG spatial information, Y is the corresponding pixels in the image. *i* and *j* represent the *j*th sample point (1 ∼ 20) of *i*th channel (1∼32). The converted images were then input to the Xception network for model training. Finally, the classification result was obtained by the SoftMax layer. In the model training and classification, fivefold cross-validation was used to improve the generalization ability of the model. Specifically, a total of 1,800 data samples extracted for each emotion state (15-participant × 6-trial × 20-s) were evenly divided into 5 parts, and the sample data were fed into the Xception network sequentially using the fivefold cross-validation method. For each type of emotion, 1,080 images were used as the training set, 360 images were used as the validation set, and 360 images were used as the test set.

#### Optimization of Channel Combination

Human cortex can be roughly divided into frontal, parietal, temporal and occipital cortexs according to the brain anatomy (Talos et al., 2006). In particular, the frontal cortex has been shown to be specifically related to emotion processing and regulation (Koelsch, 2014). Thus, except using the EEG data of entire 30 channels, we extracted a total of 8 channels in the frontal region, including Fp1, Fpz, Fp2, F7, F3, Fz, F4, F8, to assess whether using channels within the frontal cortex only is sufficient to achieve satisfactory classification performance. Moreover, we also sought to evaluate the classification performance when using only a subset of the channels in the frontal cortex.

Sequential feature selection algorithms are effective methods to select a suitable subset of features for classification (Cicalese et al., 2020). To further explore the optimal channels in the frontal cortex for emotion recognition, we applied a sequential backward selection algorithm through the following three steps.

*Step 1: Among the eight channels in the frontal cortex, we randomly selected seven channels for classification and obtained their classification performance.*

*Step 2: The channel combination with the highest classification accuracy after Step 1 were identified.*

*Step 3: Among the seven channels we identified in Step 2, we randomly selected six channels and verified the classification accuracy by repeating step 1. We repeated the step 1 and 2 by iteratively selected less channels each time until the number of selected channels reduced to two.*

#### Performance Evaluation

To quantitatively evaluate and compare the classification performance of the network, four metrics, including accuracy, sensitivity, specificity and F1 score, were adopted, as defined below:


(2) $$A\,c\,c\,u\,r\,a\,c\,y=\frac{T\,P+T\,N}{T\,P+T\,N+F\,P+F\,N}$$

(3) $$S\,e\,n\,s\,i\,t\,i\,v\,i\,t\,y\,\left(R\,e\,c\,a\,l\,l\right)=\frac{T\,P}{T\,P+F\,N}$$

(4) $$S\,p\,e\,c\,i\,f\,i\,c\,i\,t\,y=\frac{T\,N}{T\,N+F\,P}$$

$$F\,1-s\,o\,r\,c\,e=\frac{2\cdot \text{Re}\,c\,a\,l\,l\cdot \text{Pr}\,e\,c\,i\,s\,i\,o\,n}{\text{Rec}\,a\,l\,l+\text{Pr}\,e\,c\,i\,s\,i\,o\,n},w\,i\,t\,h\,\text{Pr}\,e\,s\,c\,i\,s\,i\,o\,n=\frac{T\,P}{T\,P+F\,P}$$

where TP, TN, FP, and FN were true positive, true negative, false positive, and false negative results, respectively.

#### Effect of Dynamic Functional Connectivity on Classification Performance

In addition to the investigation of spatial effect of EEG data, we also sought to explore how the time-varying alterations of functional network, constructed by channels in the frontal cortex, may affect the emotion recognition. Here, we obtained the functional connectivity (FC) between any two channels in the frontal cortex by calculating the coherence of the two single-trial time series EEG data. Coherence has been widely used to measure the level of synchronization between two physiological time series signals in previous studies (Hu et al., 2019; Li et al., 2020c; Zhang et al., 2020; Liu et al., 2022), which is mathematically calculated as:


(6) $$C\,o\,h_{x\,y}\,\left(f\right)=\frac{\left|\frac{1}{n}\,\sum_{k=1}^{n}A\,x\,\left(f,k\right)\,A\,y\,\left(f,k\right)\,e^{i\,\left(\varphi_{x}\,\left(f,k\right)-\varphi_{y}\,\left(f,k\right)\right)}\right|}{\sqrt{\frac{1}{n}\sum_{k=1}^{n}A_{\text{x}}^{2}\left(f,k\right))(\frac{1}{n}\sum_{k=1}^{n}A_{y}^{2}\left(f,k\right))}}$$

where n is the length of data, A and φ represent the amplitude and phase of the signal, respectively. The numerator term is the cross-spectral density between the two single-trial signals (x and y) at frequency f. The denominator is the square root of the product of the power spectrum of the two single-trial signals (x and y) at frequency f.

To assess how the time-varying functional network of frontal cortex may affect the classification performance, we first used the 2D coherence matrix obtained from the first trial EEG data to train the classification model and examine the performance. Then we iteratively concatenated the coherence matrix of next trial to the previous trial and repeated the classification process until all six trials were used.

## Results

### Emotion Behavior Analysis

The statistical analyses of the subjective emotional rating scores (valence and arousal) were performed using paired *t*-test, and results are shown in Table 2. There were significant differences between the two emotion states in terms of valence (*p* < 0.0001) and arousal level (*p* < 0.0001).

**TABLE 2:** Statistical analyses of the emotional behavior.

	Valence	Arousal
LVA	2.75 ± 1.17	2.98 ± 1.08
HVA	6.93 ± 1.25	7.01 ± 1.21
*p*-value (paired *t*-test)	<0.0001	<0.0001

### Classification Performance Using Electroencephalogram Channels in Frontal Cortex

Results of the classification of binary emotion states obtained from whole-head EEG signals (30-channel) and frontal EEG signals (8-channel) is summarized and shown in Table 3. Overall, classification performance using the 8 channels in the frontal region, including accuracy, sensitivity, specificity, and F1 score, was slightly better than those using all 30 EEG channels, though no statistical test was performed.

**TABLE 3:** Classification performance using whole-head EEG signal (30 channels) and frontal EEG signal (8 channels).

Channels	Accuracy (%)	Sensitivity (%)	Specificity (%)	F1 (%)
30 Channels	71.16 ± 3.67	70.84 ± 5.87	70.54 ± 2.39	71.29 ± 3.98
8 Channels	71.59 ± 2.49	71.19 ± 2.48	72.11 ± 3.26	73.82 ± 3.01

### Classification Performance Using Optimal Channel Combination in Frontal Cortex

**FIGURE 4:**
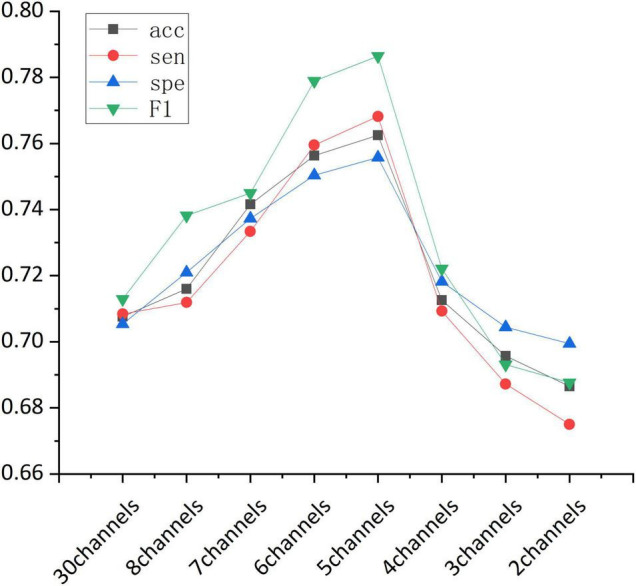
The classification performance (accuracy, sensitivity, specificity, and F1-score) obtained from different channel combinations.

Accuracy of the sequential backward selection algorithm using channels in frontal cortex is shown in Table 4. The highest classification accuracy (76.84%) was achieved using the channels including Fp1, Fpz, Fp2, F7, F8, followed by the classification accuracy (75.87%) when the selected channels were Fp1, Fpz, Fp2, F7, F3, and F8. Figure 4 shows the overall classification performance obtained from different numbers of channels in the frontal cortex. It can be observed that, the combination of 5 channels achieved the best classification results, as evidenced the peaks at accuracy, sensitivity, specificity and F1 score.

**TABLE 4:** Classification accuracy of the sequential backward selection algorithm using channels in frontal cortex.

Channels	Remove F7	Remove F8	Remove Fp2	Remove Fpz	Remove Fp1	Remove F3	Remove F4	Remove Fz
Fp1_Fpz_Fp2_F7_F3_F4_F8_Fz	72.81%	69.56%	72.36%	70.95%	71.82%	73.26%	73.26%	74.47%
Fp1_Fpz_Fp2_F7_F3_F4_F8	68.27%	67.94%	70.67%	69.52%	70.51%	75.59%	75.87%	
Fp1_Fpz_Fp2_F7_F3_F8	70.79%	72.82%	73.28%	72.83%	74.61%	76.84%		
Fp1_Fpz_Fp2_F7_F8	70.32%	70.37%	70.75%	69.66%	72.91%			
Fpz_Fp2_F7_F8	66.92%	68.26%	69.11%	70.38%				
Fp2_F7_F8	64.45%	67.56%	69.35%					

Overall, our findings suggested that, in terms of classification performance, 8 channels in the frontal cortex outperformed all the 30 channels, and the highest classification accuracy was achieved when the Fp1, Fpz, Fp2, F7, and F8 channels were combined.

As an exploratory analysis, we also evaluated the classification performance of two traditional machine learning classifiers, including Support Vector Machine (SVM) and Random Forest (RF). Based on the optimal 5-channel dataset, the mean accuracy, sensitivity, specificity, and F1-sorce achieved by SVM are 58.33, 52.10, 61.85, and 57.37%, respectively. The mean accuracy, sensitivity, specificity, and F1-sorce achieved by RF are 53.89, 52.06, 55.33, and 53.19%, respectively.

### Effect of Dynamic Functional Connectivity on Classification Performance

**FIGURE 5:**
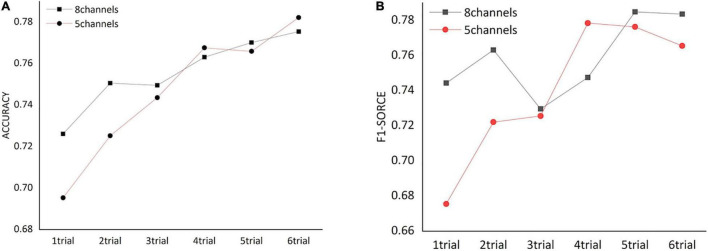
The classification performance [accuracy **(A)** and F1 score **(B)**] obtained from functional networks constructed by different numbers of trials.

We assessed how the dynamic FC of frontal cortex could affect the classification of HVA and LVA emotions by conducting a trial-by-trial classification. As shown in Figure 5, for both channel combinations, classification performance including accuracy and F1 score increased as more trials were added. Specifically, when number of trials exceeded 3, the classification accuracy and F1 score obtained from the 5-channel combination showed a relatively stable pattern.

**FIGURE 6:**
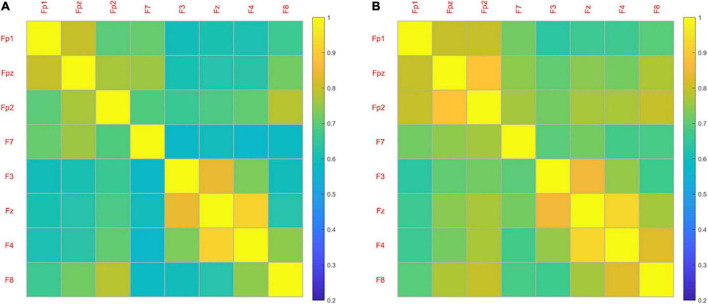
The averaged functional network of frontal cortex for both HVA **(A)** and LVA **(B)** states.

Figure 6 shows the averaged FC network of frontal cortex for both emotion states. Overall, the coherence-based functional network induced by LVA emotion demonstrated more active (higher coherence) FC among some channels compared to the functional network induced by the HVA emotion, particularly among the channels within the prefrontal cortex including Fp1, Fpz and Fp2. Statistical analysis of the averaged coherence values (Fisher’s r to z) between the two emotion states indicated that mean FC (0.29 ± 0.04) induced by LVA emotion was significantly stronger than the mean FC (0.28 ± 0.04) induced by HVA (*p* = 0.017, paired *t*-test).

## Discussion

Emotion processing and regulation plays a vital role in our daily life. Proper emotion perception serves the inherent biological function to contextualize external information, communicate with others, and help individuals cope with everyday challenges and stress. Despite the progress of emotion recognition achieved by previous studies, there remains important challenges in optimizing the study protocol of an EEG-based emotion recognition system in terms of channel configuration and presentation duration. To address the above problems, this study used the Xception neural network, combined with the spatial feature information of the EEG, to systematically evaluate the effects of musical stimuli at different valence-arousal level (high valence-arousal vs. low valence-arousal) on human emotions. The findings showed that the frontal cortex may serve as a key region for emotion recognition. An appropriate selection of EEG channels and experimental paradigm could improve the classification performance of emotion recognition system.

### Electroencephalogram Channels in the Frontal Cortex Improve Emotion Recognition

Human emotions can be conceptualized in two dimensions and classified based on their valence-arousal scale. Valence refers to happiness that generally ranges from negative to positive, while arousal indicates the activation level that ranges from low to high (Kim and Andre, 2008). Several studies have linked human emotion processing to manifestations of brain activity, particularly communication or coupling among distinct cortices of the brain. It has been shown in several EEG-based studies that EEG signals can be used to robustly detect and classify emotion states at different valence and arousal levels. In terms of emotion-related brain region, emotion processing is highly correlated with neural activity in the frontal cortex compared to other regions of the brain such as temporal, parietal, and occipital (Lin et al., 2014; Zhang et al., 2021). In particular, the left frontal area is more responsible for the processing of high valence-arousal emotions such as joy, interest, and happiness, while the right frontal region may be involved in the processing of low valence-arousal emotions such as fear, disgust, and sadness (Schmidt and Trainor, 2001). A recent study also confirmed that activity of the left frontal cortex is related to positive emotions, while negative emotions are more closely related to the right frontal cortex (Takehara et al., 2020). Following the similar idea, in this study we sought to assess the effect of EEG signals collected from the frontal region on classification of emotion states at low and high levels of valence and arousal. We showed that classification accuracy obtained from EEG channels in frontal cortex outperformed the accuracy based on the whole-head EEG channels, which is consistent with the findings reported in previous studies (Schmidt and Trainor, 2001; Lin et al., 2014; Takehara et al., 2020).

In addition, we also took a further step into the frontal cortex by assessing the optimal channels combination for emotion recognition though a sequential backward selection algorithm. Our findings indicates that emotion processing may be closely linked to specific brain regions within the frontal cortex, particularly the lateral (F7, F8) and anterior regions (Fp1, Fpz, Fp2) of the frontal cortex. According to a previous study, the lateralized EEG asymmetry between the left-right hemisphere can well characterize the changes of emotional states (Allen et al., 2004), which may partially support the findings in our study. Specifically, several studies have showed that the theta power of lateral channels such as FT7-FT8 and F7-F8 was associated with the valence and arousal scale (Aftanas et al., 2001, 2004). Since theta waves is observed during sleep and are specifically relevant to the arousal level, the identified F7 and F8 channels in our optimal channels set may mainly reflect the neural fluctuation related to arousal level in classification of emotion states. In addition, according to previous studies, Fp1 and Fp2 are found to be effective in discerning emotional states with high confidence (Yoon and Chung, 2011), wherein the left frontal (Fp1) is associated with negative emotion and the right frontal (Fp2) is associated with positive emotion (Bos, 2007; Ang et al., 2017). We found that channels in anterior part of the frontal cortex are beneficial to the emotion recognition, which is consistent with the literature. However, it should be noted that channels in the anterior part of frontal cortex, such as Fp1 and Fp2, could be substantially affected by ocular movement artifact. The effectiveness of using these channels to study emotion states remains to be tested by further studies. Despite that, a practical suggestion based on our finding is that acquiring EEG signals from regional electrodes, especially from the frontal regions, may help improve the performance of the emotion classification model as well as advance the development of low-cost EEG device with reliable performance.

### Dynamic Functional Network of Frontal Cortex Affects Emotion Recognition

A majority of EEG-based studies of emotion recognition have mainly adopted analyses at the single-electrode level. However, as previously mentioned, emotion processing is a complex process that involves active interactions among different brain regions. We argue that EEG-based classification and recognition of different emotion states may be more valuable if EEG measurements could be analyzed at a network-based level rather than being based simply on analyses at the independent electrode level. By using the coherence-based functional network as input features, we showed that the frontal network could achieve better classification performance compared to the performance using EEG temporal series in the frontal cortex, with both accuracy and F1 score exceeded 76%. This finding supports the premise that emotional states might be characterized by unique patterns of EEG-based functional connectivity, which is also in line with conclusions of previous studies (Lee and Hsieh, 2014). Moreover, classification using dynamic trial-by-trial FC networks suggested that performance of emotion recognition was positively correlated with the number of trials. Also, the enhancement of classification performance became quite stable when the trial number exceeds 3. This indicates that the functional network induced by an emotion state may be adaptive in a time-varying manner, and such adaptation would remain stable even more emotional stimuli is administrated. Our finding here, together with the findings from the spatially optimized EEG channel combination (see section “Classification Performance Using Electroencephalogram Channels in Frontal Cortex”), provides a new perspective for optimizing study design when conducting neuroimaging-based emotion processing and regulation studies.

We found the functional network induced by LVA emotion demonstrated more active (higher coherence) FC than the one induced by the HVA emotion. Previous studies have shown that different functional connectivity patterns may be induced by different emotional states. Several studies reported that coherence of the brain network induced by low arousal stimuli was greater compared to that induced by high arousal emotional stimuli (Holczberger et al., 2012), possibly due to a more stable brain synchronization at low arousal state. The results of our study agreed with the findings in these studies; HVA emotion demonstrated a lower frontal network compared to the LVA emotion. Similar evidence was reported when participants were watching stressful vs. enjoyable film sequences (Schellberg et al., 1990). However, previous studies also proposed that high arousal emotion showed a greater strength of brain network than the low arousal (Miskovic and Schmidt, 2010; Cao et al., 2020). These divergent findings might be due to differences in the essence of connectivity measures, or the emotional stimuli used in these studies. Further exploratory studies are needed to resolve such inconsistencies.

### Limitations

This study holds several limitations that provide us with future research directions. First, mental fatigue and the degree of investment in research tasks may affect the reproducibility of EEG measurements (Shenoy et al., 2006; Ahn et al., 2016). Besides, the two types of music clips adopted to induce LVA and HVA emotions differed to each other in magnitude of the power spectrum (Figure 2A), which might potentially cause distinct brain response. As indicated in previous studies, however, different types of stimuli (e.g., music, picture viewing, facial expression) may lead to different brain response. Also, in this study we only investigated emotion at two distinct levels (i.e., low valence-arousal and high valence-arousal), which did not cover more emotion states (e.g., high valence and low arousal, low valence and high arousal). Therefore, comprehensive research may be needed in the future to systematically evaluate the optimal protocol and effectiveness of EEG-based emotional recognition studies. Moreover, although we used coherence as a FC measure to study the dynamic functional network induced by emotion, other multivariate methods such as phase lag index (PLI) (Li R. et al., 2019; Li et al., 2020a) and partial directed coherence (PDC) (Astolfi et al., 2007) are commonly used to establish the brain functional network. Further studies of whether such measures can be used as indices for emotion recognition will be needed. Finally, in this study we specifically focused on the role of frontal cortex in emotion recognition. However, other brain regions, such as central or parietal areas, may also serve as key hubs for emotion processing (Heller et al., 1997; Suhaimi et al., 2020). In this context, it has been showed that increased theta power in parietal area is linked to high arousal (Aftanas et al., 2002). Previous studies also suggested that brain-emotion relationship could be characterized by complex network interactions with more fine-grained spatiotemporal resolution (Nguyen et al., 2019; Fang et al., 2020). In sum, the optimal measurement protocol for EEG-based emotion studies remains to be determined in future studies.

## Conclusion

This study presented an EEG-based emotion recognition system to classify emotion states at high valence-arousal and low valence-arousal, respectively. Through a sequential backward selection algorithm and a deep learning neural network, we showed that region-specific neuronal activity in the frontal cortex, as measured by a subset EEG channels, could improve the performance of the emotion recognition system. In addition, we also showed that the dynamic functional network within the frontal cortex may affect the classification performance of emotion states in a time-varying manner. Our findings could provide a new perspective for the development of EEG-based emotional recognition systems.

## Data Availability Statement

The raw data supporting the conclusions of this article will be made available by the authors, without undue reservation.

## Ethics Statement

The studies involving human participants were reviewed and approved by the Institutional Review Board of Nanchang Hangkong University. The patients/participants provided their written informed consent to participate in this study.

## Author Contributions

JL and LS designed the research and developed the method, and analyzed the data with the support of JL, MH, and YX. JL and LS wrote the first draft of the manuscript. RL directed the study. All authors participated in the scientific discussion, revised and approved the first draft of the manuscript.

## Conflict of Interest

The authors declare that the research was conducted in the absence of any commercial or financial relationships that could be construed as a potential conflict of interest.

## Publisher’s Note

All claims expressed in this article are solely those of the authors and do not necessarily represent those of their affiliated organizations, or those of the publisher, the editors and the reviewers. Any product that may be evaluated in this article, or claim that may be made by its manufacturer, is not guaranteed or endorsed by the publisher.

## References

[B1] Aftanas L. Varlamov A. Pavlov S. Makhnev V. Reva N. (2001). Event-related synchronization and desynchronization during affective processing: emergence of valence-related time-dependent hemispheric asymmetries in theta and upper alpha band. *Int. J. Neurosci.* 110 197–219. 10.3109/00207450108986547 11912870

[B2] Aftanas L. I. Reva N. V. Varlamov A. A. Pavlov S. V. Makhnev V. P. (2004). Analysis of evoked EEG synchronization and desynchronization in conditions of emotional activation in humans: temporal and topographic characteristics. *Neurosci. Behav. Physiol.* 34 859–867. 10.1023/b:neab.0000038139.39812.eb 15587817

[B3] Aftanas L. I. Varlamov A. A. Pavlov S. V. Makhnev V. P. Reva N. V. (2002). Time-dependent cortical asymmetries induced by emotional arousal: EEG analysis of event-related synchronization and desynchronization in individually defined frequency bands. *Int. J. Psychophysiol.* 44 67–82. 10.1016/s0167-8760(01)00194-5 11852158

[B4] Ahirwal M. K. Kose M. R. (2019). Audio-visual stimulation based emotion classification by correlated EEG channels. *Health Technol.* 10 7–23. 10.1007/s12553-019-00394-5

[B5] Ahn S. Nguyen T. Jang H. Kim J. G. Jun S. C. (2016). Exploring neuro-physiological correlates of drivers’ mental fatigue caused by sleep deprivation using simultaneous EEG, ECG, and fNIRS Data. *Front. Hum. Neurosci.* 10:219. 10.3389/fnhum.2016.00219 27242483 PMC4865510

[B6] Allen J. J. Coan J. A. Nazarian M. (2004). Issues and assumptions on the road from raw signals to metrics of frontal EEG asymmetry in emotion. *Biol. Psychol.* 67 183–218. 10.1016/j.biopsycho.2004.03.007 15130531

[B7] Al-Sheikh B. Salman M. S. Eleyan A. Alboon S. (2019). Non-invasive fetal ECG extraction using discrete wavelet transform recursive inverse adaptive algorithm. *Technol. Health Care* 28 507–520. 10.3233/THC-191948 31904000

[B8] Ang A. Q.-X. Yeong Y. Q. Wee W. (2017). Emotion classification from EEG signals using time-frequency-DWT features and ANN. *J. Comp. Commun.* 5 75–79. 10.4236/jcc.2017.53009

[B9] Astolfi L. Cincotti F. Mattia D. Marciani M. G. Baccala L. A. de Vico Fallani F. (2007). Comparison of different cortical connectivity estimators for high-resolution EEG recordings. *Hum. Brain Mapp.* 28 143–157. 10.1002/hbm.20263 16761264 PMC6871398

[B10] Bo H. Ma L. Liu Q. Xu R. Li H. (2018). Music-evoked emotion recognition based on cognitive principles inspired EEG temporal and spectral features. *Int. J. Mach. Learn. Cybernet.* 10 2439–2448. 10.1007/s13042-018-0880-z

[B11] Bos D. P.-O. (2007). EEG-based emotion recognition the influence of visual and auditory stimuli. *World J. Neurosci.* 2:4.

[B12] Cao R. Hao Y. Wang X. Gao Y. Shi H. Huo S. (2020). EEG functional connectivity underlying emotional valance and arousal using minimum spanning trees. *Front. Neurosci.* 14:355. 10.3389/fnins.2020.00355 32457566 PMC7222391

[B13] Cicalese P. A. Li R. Ahmadi M. B. Wang C. Francis J. T. Selvaraj S. (2020). An EEG-fNIRS hybridization technique in the four-class classification of alzheimer’s disease. *J. Neurosci. Methods* 336:108618. 10.1016/j.jneumeth.2020.108618 32045572 PMC7376762

[B14] Delorme A. Makeig S. (2004). EEGLAB: an open source toolbox for analysis of single-trial EEG dynamics including independent component analysis. *J. Neurosci. Methods* 134 9–21. 10.1016/j.jneumeth.2003.10.009 15102499

[B15] Fang F. Potter T. Nguyen T. Zhang Y. (2020). Dynamic reorganization of the cortical functional brain network in affective processing and cognitive reappraisal. *Int. J. Neural. Syst.* 30:2050051. 10.1142/S0129065720500513 32812469

[B16] Gupta V. Chopda M. D. Pachori R. B. (2019). Cross-subject emotion recognition using flexible analytic wavelet transform From EEG Signals. *IEEE Sens. J.* 19 2266–2274. 10.1109/jsen.2018.2883497

[B17] Hasanzadeh F. Annabestani M. Moghimi S. (2021). Continuous emotion recognition during music listening using EEG signals: a fuzzy parallel cascades model. *Appl. Soft Comp.* 101:107028. 10.1016/j.asoc.2020.107028

[B18] Heller W. Nitschke J. B. Lindsay D. L. (1997). Neuropsychological correlates of arousal in self-reported emotion. *Cogn. Emot.* 11 383–402. 10.1080/026999397379854

[B19] Holczberger E. M. Bernal J. Silva J. Yañez G. Rodríguez M. Prieto B. (2012). Electroencephalographic coherences during emotion identification task. *World J. Neurosci.* 2 248–253. 10.4236/wjns.2012.24037

[B20] Hossen A. Deuschl G. Groppa S. Heute U. Muthuraman M. (2020). Discrimination of physiological tremor from pathological tremor using accelerometer and surface EMG signals. *Technol. Health Care* 28 461–476. 10.3233/THC-191947 32280070

[B21] Hu Y. Zhang Q. Li R. Potter T. Zhang Y. (2019). “Graph-based Brain Network Analysis in Epilepsy: an EEG Study,” in *2019 9th International IEEE/EMBS Conference on Neural Engineering (NER)* (Piscataway: IEEE), 130–133.

[B22] Jenke R. Peer A. Buss M. (2014). Feature extraction and selection for emotion recognition from EEG. *IEEE Trans. Affect. Comp.* 5 327–339. 10.1109/taffc.2014.2339834

[B23] Kim J. Andre E. (2008). Emotion recognition based on physiological changes in music listening. *IEEE Trans. Patt. Anal. Mach. Intell.* 30 2067–2083. 10.1109/TPAMI.2008.26 18988943

[B24] Koelsch S. (2014). Brain correlates of music-evoked emotions. *Nat. Rev. Neurosci.* 15 170–180. 10.1038/nrn3666 24552785

[B25] Kulke L. Feyerabend D. Schacht A. (2020). A comparison of the affectiva iMotions facial expression analysis software with EMG for identifying facial expressions of emotion. *Front. Psychol.* 11:329. 10.3389/fpsyg.2020.00329 32184749 PMC7058682

[B26] Lee Y. Y. Hsieh S. (2014). Classifying different emotional states by means of EEG-based functional connectivity patterns. *PLoS One* 9:e95415. 10.1371/journal.pone.0095415 24743695 PMC3990628

[B27] Li P. Liu H. Si Y. Li C. Li F. Zhu X. (2019). EEG based emotion recognition by combining functional connectivity network and local activations. *IEEE Trans. Biomed. Eng.* 66 2869–2881. 10.1109/TBME.2019.2897651 30735981

[B28] Li R. Li S. Roh J. Wang C. Zhang Y. (2020a). Multimodal neuroimaging using concurrent EEG/fNIRS for poststroke recovery assessment: an exploratory study. *Neurorehabil. Neural. Repair.* 34 1099–1110. 10.1177/1545968320969937 33190571

[B29] Li R. Nguyen T. Potter T. Zhang Y. (2019). Dynamic cortical connectivity alterations associated with Alzheimer’s disease: an EEG and fNIRS integration study. *Neuroimage. Clin.* 21:101622. 10.1016/j.nicl.2018.101622 30527906 PMC6411655

[B30] Li R. Potter T. Huang W. Zhang Y. (2017). Enhancing performance of a hybrid EEG-fNIRS system using channel selection and early temporal features. *Front. Human Neurosci.* 11:462. 10.3339/Fnhum.2017.00462 PMC5605645 28966581

[B31] Li R. Zhao C. Wang C. Wang J. Zhang Y. (2020b). Enhancing fNIRS analysis using EEG rhythmic signatures: an EEG-Informed fNIRS analysis study. *IEEE Trans. Biomed. Eng.* 67 2789–2797. 10.1109/TBME.2020.2971679 32031925

[B32] Li X. La R. Wang Y. Hu B. Zhang X. (2020c). A deep learning approach for mild depression recognition based on functional connectivity using electroencephalography. *Front. Neurosci.* 14:192. 10.3389/fnins.2020.00192 32300286 PMC7142271

[B33] Liliana D. Y. (2019). Emotion recognition from facial expression using deep convolutional neural network. *J. Phys. Confer. Ser.* 1193:12004. 10.1088/1742-6596/1193/1/012004

[B34] Lin Y. P. Wang C. H. Jung T. P. Wu T. L. Jeng S. K. Duann J. R. (2010). EEG-based emotion recognition in music listening. *IEEE Trans. Biomed. Eng.* 57 1798–1806. 10.1109/TBME.2010.2048568 20442037

[B35] Lin Y. P. Yang Y. H. Jung T. P. (2014). Fusion of electroencephalographic dynamics and musical contents for estimating emotional responses in music listening. *Front. Neurosci.* 8:94. 10.3389/fnins.2014.00094 24822035 PMC4013455

[B36] Liu H. Gao Y. Huang W. Li R. Houston M. Benoit J. S. (2022). Inter-muscular coherence and functional coordination in the human upper extremity after stroke. *Math. Biosci. Eng.* 19 4506–4525. 10.3934/mbe.2022208 35430825

[B37] Liu J. Yuan C. Sun X. Sun L. Dong H. Peng Y. (2021). The measurement of Cobb angle based on spine X-ray images using multi-scale convolutional neural network. *Phys. Eng. Sci. Med.* 44 809–821. 10.1007/s13246-021-01032-z 34251603

[B38] Liu Z. Wu M. Cao W. Chen L. Xu J. Zhang R. (2017). A facial expression emotion recognition based human-robot interaction system. *IEEE/CAA J. Automat. Sin.* 4 668–676. 10.1109/jas.2017.7510622

[B39] Mauss I. B. Robinson M. D. (2009). Measures of emotion: a review. *Cogn. Emot.* 23 209–237. 10.1080/02699930802204677 19809584 PMC2756702

[B40] Miskovic V. Schmidt L. A. (2010). Cross-regional cortical synchronization during affective image viewing. *Brain Res.* 1362 102–111. 10.1016/j.brainres.2010.09.102 20920492

[B41] Namazi H. Aghasian E. Ala T. S. (2020). Complexity-based classification of EEG signal in normal subjects and patients with epilepsy. *Technol. Health Care* 28 57–66. 10.3233/THC-181579 31104032

[B42] Nguyen T. Zhou T. Potter T. Zou L. Zhang Y. (2019). The cortical network of emotion regulation: insights from advanced EEG-fMRI Integration Analysis. *IEEE Trans. Med. Imag.* 38 2423–2433. 10.1109/TMI.2019.2900978 30802854

[B43] Noroozi F. Corneanu C. A. Kaminska D. Sapinski T. Escalera S. Anbarjafari G. (2021). Survey on emotional body gesture recognition. *IEEE Trans. Affect. Comp.* 12 505–523. 10.1109/taffc.2018.2874986

[B44] Peng G. Dong H. Liang T. Li L. Liu J. (2021). Diagnosis of cervical precancerous lesions based on multimodal feature changes. *Comput. Biol. Med.* 130:104209. 10.1016/j.compbiomed.2021.104209 33440316

[B45] Raghu S. Sriraam N. Temel Y. Rao S. V. Kubben P. L. (2020). EEG based multi-class seizure type classification using convolutional neural network and transfer learning. *Neural. Netw.* 124 202–212. 10.1016/j.neunet.2020.01.017 32018158

[B46] Sarkar P. Etemad A. (2021). Self-supervised ECG representation learning for emotion recognition. *IEEE Trans. Affect. Comp.* 2021:842. 10.1109/taffc.2020.3014842

[B47] Sarno R. Munawar M. N. Nugraha B. T. Sarno R. Munawar M. Nugraha B. (2016). Real-time electroencephalography-based emotion recognition system. *Int. Rev. Comput. Softw.* 11 456–465. 10.15866/irecos.v11i5.9334

[B48] Schellberg D. Besthorn C. Klos T. Gasser T. (1990). EEG power and coherence while male adults watch emotional video films. *Int. J. Psychophysiol.* 9 279–291. 10.1016/0167-8760(90)90060-q 2276946

[B49] Schmidt L. A. Trainor L. J. (2001). Frontal brain electrical activity (EEG) distinguishes valence and intensity of musical emotions. *Cogn. Emot.* 15 487–500. 10.1080/02699930126048

[B50] Shenoy P. Krauledat M. Blankertz B. Rao R. P. Muller K. R. (2006). Towards adaptive classification for BCI. *J. Neural. Eng.* 3 R13–R23. 10.1088/1741-2560/3/1/R02 16510936

[B51] Song T. Zheng W. Song P. Cui Z. (2020). EEG emotion recognition using dynamical graph convolutional neural networks. *IEEE Trans. Affect. Comp.* 11 532–541. 10.1109/taffc.2018.2817622

[B52] Suhaimi N. S. Mountstephens J. Teo J. (2020). EEG-based emotion recognition: a state-of-the-art review of current trends and opportunities. *Comput. Intell. Neurosci.* 2020:8875426. 10.1155/2020/8875426 33014031 PMC7516734

[B53] Takehara H. Ishihara S. Iwaki T. (2020). Comparison between facilitating and suppressing facial emotional expressions using frontal EEG Asymmetry. *Front. Behav. Neurosci.* 14:554147. 10.3389/fnbeh.2020.554147 33192362 PMC7581785

[B54] Talos I. F. Mian A. Z. Zou K. H. Hsu L. Goldberg-Zimring D. Haker S. (2006). Magnetic resonance and the human brain: anatomy, function and metabolism. *Cell Mol. Life Sci.* 63 1106–1124. 10.1007/s00018-005-5522-4 16568243 PMC11136333

[B55] Tang X. Zhao J. Fu W. (2019). “Research on extraction and classification of EEG features for multi-class motor imagery,” in *2019 IEEE 4th Advanced Information Technology, Electronic and Automation Control Conference (IAEAC)*, Piscataway. 693–697.

[B56] Taran S. Bajaj V. (2019). Emotion recognition from single-channel EEG signals using a two-stage correlation and instantaneous frequency-based filtering method. *Comput. Methods Prog. Biomed.* 173 157–165. 10.1016/j.cmpb.2019.03.015 31046991

[B57] Wang Q. Li Y. Liu X. (2018). The influence of photo elements on EEG signal recognition. *EURASIP J. Image Video Proc.* 2018:134. 10.1186/s13640-018-0367-6

[B58] Wang X. Chen X. Cao C. (2020). Human emotion recognition by optimally fusing facial expression and speech feature. *Signal Proc. Image Commun.* 84:115831. 10.1016/j.image.2020.115831

[B59] Xu G. Shen X. Chen S. Zong Y. Zhang C. Yue H. (2019). A deep transfer convolutional neural network framework for EEG signal classification. *IEEE Access* 7 112767–112776. 10.1109/access.2019.2930958

[B60] Yao D. Qin Y. Hu S. Dong L. Bringas Vega M. L. Valdes Sosa P. A. (2019). Which reference should we use for EEG and ERP practice? *Brain Topogr.* 32 530–549. 10.1007/s10548-019-00707-x 31037477 PMC6592976

[B61] Yoon H. J. Chung S. Y. (2011). “EEG spectral analysis in valence and arousal dimensions of emotion,” in *2011 11th International Conference on Control, Automation and Systems* (Piscataway: IEEE), 1319–1322.

[B62] Zhang M. Li Z. Wang L. Yang S. Zou F. Wang Y. (2021). The resting-state electroencephalogram microstate correlations with empathy and their moderating effect on the relationship between empathy and disgust. *Front. Hum. Neurosci.* 15:626507. 10.3389/fnhum.2021.626507 34262440 PMC8273331

[B63] Zhang Q. Hu Y. Potter T. Li R. Quach M. Zhang Y. (2020). Establishing functional brain networks using a nonlinear partial directed coherence method to predict epileptic seizures. *J. Neurosci. Methods* 329:108447. 10.1016/j.jneumeth.2019.108447 31614163

[B64] Zinchenko A. Obermeier C. Kanske P. Schroger E. Villringer A. Kotz S. A. (2017). The influence of negative emotion on cognitive and emotional control remains intact in aging. *Front. Aging Neurosci.* 9:349. 10.3389/fnagi.2017.00349 29163132 PMC5671981

